# A Smartphone App to Increase Immunizations in the Pediatric Solid Organ Transplant Population: Development and Initial Usability Study

**DOI:** 10.2196/32273

**Published:** 2022-01-13

**Authors:** Amy G Feldman, Susan Moore, Sheana Bull, Megan A Morris, Kumanan Wilson, Cameron Bell, Margaret M Collins, Kathryn M Denize, Allison Kempe

**Affiliations:** 1 Adult and Child Consortium for Health Outcomes Research and Delivery Science Aurora, CO United States; 2 University of Colorado School of Medicine Aurora, CO United States; 3 Children's Hospital Colorado Aurora, CO United States; 4 Colorado School of Public Health Aurora, CO United States; 5 Bruyere and Ottawa Hospital Research Institutes The Ottawa Hospital University of Ottawa Ottawa, ON Canada; 6 CANImmunize Inc Ottawa, ON Canada

**Keywords:** vaccinations, transplantation, mobile app, agile development, immunization, mHealth, mobile health, children, transplant recipients, pediatric transplant recipients, pediatrics

## Abstract

**Background:**

Vaccine-preventable infections result in significant morbidity, mortality, and costs in pediatric transplant recipients. However, at the time of transplant, less than 20% of children are up-to-date for age-appropriate immunizations that could prevent these diseases. Smartphone apps have the potential to increase immunization rates through their ability to provide vaccine education, send vaccine reminders, and facilitate communication between parents and a multidisciplinary medical group.

**Objective:**

The aim of this study was to describe the development of a smartphone app, Immunize PediatricTransplant, to promote pretransplant immunization and to report on app functionality and usability when applied to the target population.

**Methods:**

We used a mixed methods study design guided by the Mobile Health Agile Development and Evaluation Lifecycle. We first completed a formative research including semistructured interviews with transplant stakeholders (12 primary care physicians, 40 parents or guardians of transplant recipients, 11 transplant nurse coordinators, and 19 transplant subspecialists) to explore the acceptability of an immunization app to be used in the pretransplant period. Based on these findings, CANImmunize Inc developed the Immunize PediatricTransplant app. We next held 2 focus group discussions with 5-6 transplant stakeholders/group (n=11; 5 parents of transplant recipients, 2 primary care physicians, 2 transplant nurse coordinators, and 2 transplant subspecialists) to receive feedback on the app. After the app modifications were made, alpha testing was conducted on the functional prototype. We then implemented beta testing with 12 stakeholders (6 parents of transplant recipients, 2 primary care doctors, 2 transplant nurse coordinators, and 2 transplant subspecialists) to refine the app through an iterative process. Finally, the stakeholders completed the user version of the Mobile Application Rating Scale (uMARS) to assess the functionality and quality of the app.

**Results:**

A new Android- and Apple-compatible app, Immunize PediatricTransplant, was developed to improve immunization delivery in the pretransplant period. The app contains information about vaccine use in the pretransplant period, houses a complete immunization record for each child, includes a communication tool for parents and care providers, and sends automated reminders to parents and care providers when immunizations are due. During usability testing, the stakeholders were able to enter a mock vaccine record containing 16 vaccines in an average of 8.1 minutes (SD 1.8) with 87% accuracy. The stakeholders rated engagement, functionality, aesthetics, and information quality of the app as 4.2/5, 4.5/5, 4.6/5, and 4.8/5, respectively. All participants reported that they would recommend this app to families and care teams with a child awaiting solid organ transplant.

**Conclusions:**

Through a systematic, user-centered, agile, iterative approach, the Immunize PediatricTransplant app was developed to improve immunization delivery in the pretransplant period. The app tested well with end users. Further testing and agile development among patients awaiting transplant are needed to understand real-world acceptability and effectiveness in improving immunization rates in children awaiting transplant.

## Introduction

Due to lifelong immunosuppression, solid organ transplant recipients are at increased risk of life-threatening infections [1-3]. Vaccine-preventable infections (VPIs) occur in up to 15% of pediatric solid organ transplant recipients in the first 5 years posttransplant, a rate of up to 87 times higher than in the general pediatric population [4,5]. These VPIs result in lengthy hospitalizations, morbidity, and mortality. In addition, VPIs can increase the cost of transplantation by US $120,000 [4,5]. To prevent these infections, it is crucial for transplant candidates to receive all age-appropriate vaccines in the pretransplant period. However, less than 20% of pediatric liver transplant recipients are up to date on age-appropriate immunizations at the time of transplant [6]. In a recent qualitative study with 82 transplant stakeholders (including transplant hepatologists, nephrologists, cardiologists, infectious diseases physicians, transplant nurse coordinators, primary care physicians, and the parents of transplant recipients), the following barriers to pretransplant immunization were identified: (1) gaps in knowledge about the timing and safety of pretransplant vaccines; (2) lack of communication, coordination, and follow-up between team members regarding immunizations; (3) difficulty remembering when vaccines were due; and (4) lack of a centralized immunization record that could be easily accessible by all team members [7].

While human resources are an important component in the overall strategy to address immunization rates, provider-driven interventions (ie, phone call reminders and individually created calendar reminders) are expensive and difficult to sustain. Health information technology tools, including health care mobile apps for use on a personal smartphone or desktop computer, have been demonstrated to be a sustainable strategy for facilitating patient provider communication, disseminating high-quality evidence-based information to end users on a global scale, increasing adherence to medical regimens and tracking when medical interventions are due, and improving outcomes in chronic illnesses [8-12]. mHealth (mobile health) apps have been successfully created and implemented to facilitate immunization delivery [13-20]. However, mHealth apps have never been utilized to improve immunization rates in high-risk populations (such as children awaiting transplant) who require a tailored vaccine schedule (such as the accelerated vaccine schedule) and comanagement by multiple providers (including the primary care physician and transplant team) who in turn may operate on different electronic medical records (EMRs). The goal of this study was to describe the development of a smartphone app, Immune PediatricTransplant, to promote pretransplant immunization and to report on app functionality and usability when piloted in the target population.

## Methods

### mHealth Agile Development and Evaluation Lifecycle

**Figure 1:**
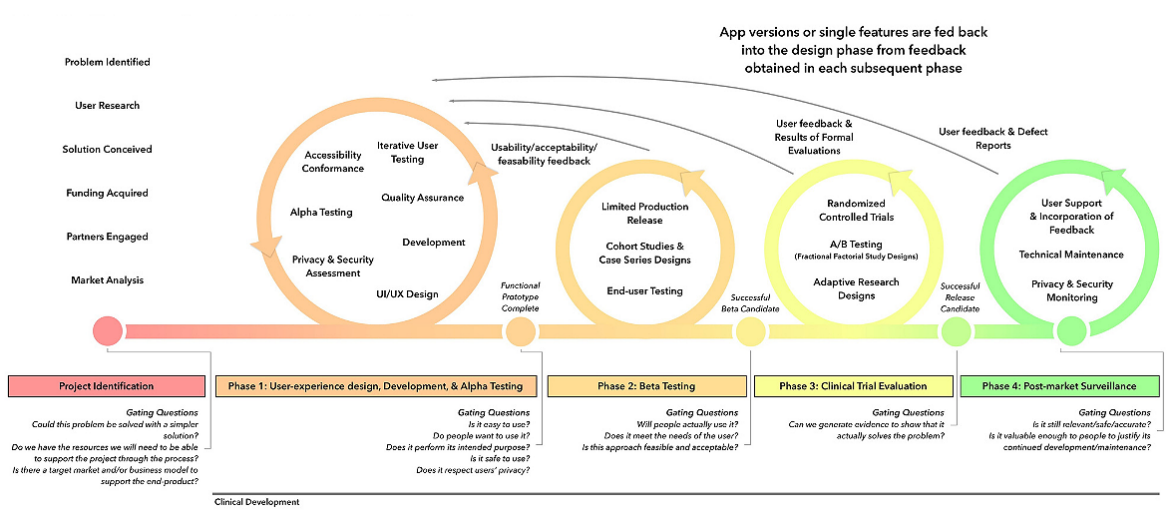
mHealth Agile Development and Evaluation Lifecycle (adapted from Wilson et al [21]). UI: user interface; UX: user experience.

The mHealth Agile Development and Evaluation Lifecycle guided this study. This lifecycle focuses on safety and efficacy while also allowing for rapid and iterative development and evaluation that is required to create high-quality, effective, thoroughly tested, evidence-based digital tools. The mHealth Agile Development and Evaluation Lifecycle is divided into 5 stages: phase 0—project identification; phase 1—user experience, design, development, and alpha testing; phase 2—beta testing; phase 3—clinical trial evaluation; and phase 4—postmarket surveillance [21] (Figure 1). This study focused on the first 3 phases of this cycle—project identification; user experience design, development, and alpha testing; and beta testing.

### Approach and Recruitment

**Figure 2:**
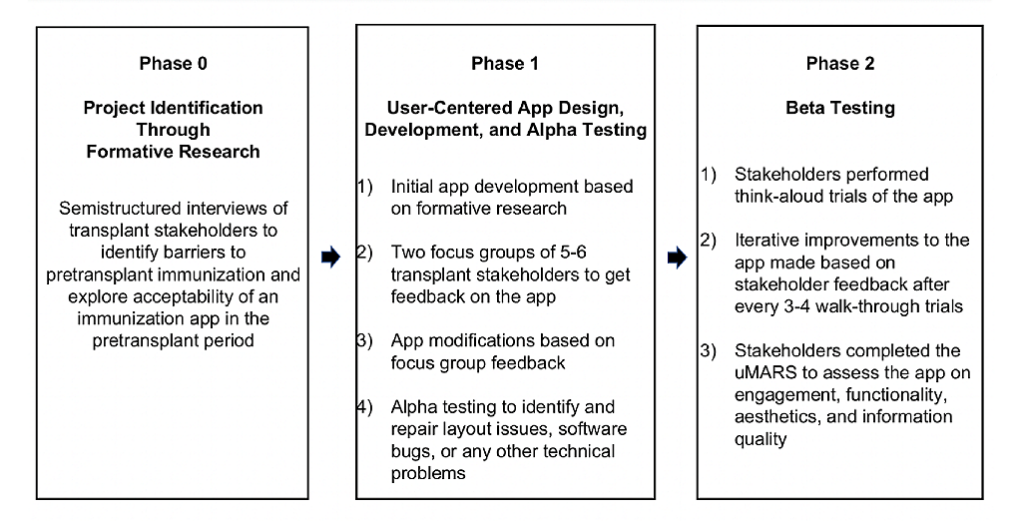
Development process of the Immunize PediatricTransplant app. uMARS: user version of the Mobile Application Rating Scale.

We used a sequential user-centered design process approach focusing on the first 3 phases of the mHealth Agile Development and Evaluation Lifecycle (Figure 2). All English-speaking transplant hepatologists, cardiologists, nephrologists, infectious diseases physicians, primary care physicians, and parents of children who received liver, heart, and kidney transplants between January 1, 2011, and August 30, 2019, at the Children’s Hospital Colorado (CHCO), Ann & Robert H. Lurie Children’s Hospital of Chicago (Lurie) and Children’s Hospital of Philadelphia (CHOP) were invited to participate. Health care providers were invited to participate via an email invitation. The parents of transplant recipients were approached in clinic or were sent a written invitation. The participants received a reimbursement for participating in the study (US $10 per interview, US $150 per focus group, and US $150 per think-aloud walk-through app trial testing). Institutional review board approval was obtained from the University of Colorado (all research took place through the University of Colorado; CHOP and Lurie only assisted with recruitment), and verbal informed consent was obtained by the interviewer before starting the interview, focus group or think-aloud walk-through app trial [7].

### Phase 0: Project Identification Through Formative Research to Understand the Barriers to Pretransplant Immunization and Assess Acceptability of a Pretransplant Immunization App

To develop an evidence and theory-based app, we identified barriers to pretransplant immunization based on relevant literature, prior research by our group, and the domains from the Theoretic Domains Framework [4-6,22-26]. We conducted semistructured interviews with 82 transplant stakeholders (including the parents of transplant recipients, transplant nurse coordinators, transplant infectious diseases physicians, primary care physicians, and transplant nephrologists, cardiologists, and hepatologists) to identify barriers to pretransplant immunization and to explore whether an immunization app would be useful in addressing these barriers. We used a team-based inductive approach to analyze the results [7]. Our focus of this manuscript is to present new findings regarding use and acceptability of an immunization app.

### Phase 1: User-Centered App Design, Development, and Alpha Testing

Based on the formative research, we worked with CANImmunize Inc to develop a new app for Apple and Android smartphones. The content of the app targets immunization barriers identified in Step 1. Specifically, the app (1) incorporates information about pretransplant vaccine safety and timing; (2) houses an easily accessible cloud-based immunization record for each child; (3) includes a chat or communication feature for providers and parents to communicate about immunizations; and (4) provides reminders for parents and providers when vaccines are due based on the accelerated Centers for Disease Control and Prevention vaccine schedule for transplant candidates.

We held 2 focus group discussions with 5 transplant stakeholder participants in the first group and 6 transplant stakeholder participants in the second group (N=11; 5 parents of transplant recipients, 2 primary care physicians, 2 transplant nurse coordinators, and 2 hepatologists) to obtain feedback on the initial version of the app. We implemented a focus group protocol and semistructured discussion guide consistent with the recommended focus group methodology [27]. We showed the participants screenshots from the app and asked for their thoughts. Both focus groups were led by a moderator, which was held in English and lasted 60 minutes. With the permission of the participants, the focus groups were videotaped, and notes were taken. Based on the findings from the focus groups, we made modifications to the app. Upon completion of the initial functional prototype, CANImmunize Inc completed alpha testing to identify and repair any layout issues, software bugs, or other technical problems.

### Phase 2: Beta Testing

We invited 12 transplant stakeholders (6 parents of transplant recipients, 2 primary care physicians, 2 transplant nurse coordinators, and 2 hepatologists) to perform a lab usability testing of the app. Once they consented, the participants downloaded and trialed the app during a think-aloud walk-through over Zoom. Think-aloud walk-throughs are a standard approach for software development and app testing [28-30]. We asked each of the 12 stakeholders to set up an account, create a new child record, input a 16-vaccine immunization record provided by the research team, connect with the research physician, and utilize various app features (eg, including enabling touch/face ID, setting up notifications to be received via text message, and reading through the informational features of the app). This database captured all information inputted into the app and saved the time stamp at which each change was made to the user’s data. This was used to assess how long the participant spent entering the complete vaccine record, the accuracy with which they entered the vaccine record, and the stakeholder’s success in connecting with the research physician. After each participant completed the testing, we asked them to independently complete the user version of the Mobile Application Rating System (uMARS), a validated tool for end users to assess the quality of mHealth apps. The uMARS is a 26-item measure that includes subscales to assess engagement, functionality, aesthetics, and information quality of the app [31]. After every 3-4 usability tests, we incorporated feedback into a new version of the app.

## Results

### Approach and Recruitment

We interviewed 82 stakeholders including parents or guardians of heart, liver, and kidney transplant recipients, primary care physicians who took care of transplant recipients, transplant infectious diseases physicians transplant nurse coordinators, and transplant hepatologists, nephrologists, and cardiologists (Table 1).

**Table 1:** Participant demographic characteristics (N=82).

Characteristics		Values
Transplant subspecialist, n (%)		16 (20)
Transplant ID^a^ physician, n (%)		3 (4)
Transplant nurse coordinator, n (%)		11 (13)
Primary care provider, n (%)		12 (15)
Parent or guardian, n (%)		40 (49)
**Transplant center, n (%)**		
	Children’s Hospital Colorado	35 (43)
	Children’s Hospital of Philadelphia	27 (33)
	Ann and Robert H. Lurie Children’s Hospital	20 (24)
**Gender, n (%)**		
	Female	68 (83)
	Male	14 (17)
**Years in practice,^b^ n (%)**		
	0-5	10 (24)
	6-10	6 (14)
	11-20	10 (24)
	Over 20 years	16 (38)
**Interview venue, n (%)**		
	Hospital (office or conference meeting room)	18 (22)
	Telephone	64 (78)

^a^ID: infectious diseases.

^b^excluding parents (n=42).

### Phase 0: Project Identification Through Formative Research to Understand the Barriers to Pretransplant Immunization and Assess Acceptability of a Pretransplant Immunization App

Despite being from diverse geographic regions and having experience with different types of organ transplant (heart, liver, and kidney), when asked about feasibility and potential benefit, 80/82 (98%) participants believed that a mobile health app would be useful to help address and overcome these immunization barriers in the pretransplant period. Parent participants commented that an immunization app could provide educational material on “how vaccines work and what diseases they prevent.” All participants reported that a health information technology tool could improve communication by “getting everyone on the same page, especially when different providers used different EMRs.” Provider participants emphasized that having easy access to a centralized immunization record would “be great, especially for out of state patients whose information is not in the state immunization information system.” Finally, parent and provider participants stated that automated vaccine reminders “would be a huge help in reminding them to get needed vaccines” (Table 2).

**Table 2:** Selective illustrative quotations about how a transplant-specific immunization app might help address immunization barriers in the pretransplant period

Theme	Illustrative Quotation
Increase factual knowledge	I don’t know a lot of the scientific words of what the doctor says—if the app could explain the vaccine, it would be amazing. [parent] It would be great to have information about what the vaccine is, what it protects against, how often it needs to be given, and why its extra important for a soon-to-be immunocompromised child. [parent] I’d like a place to verify vaccine information; I don’t want to mess it up. [primary care provider] If there was a tool where I could enter the child’s age, the vaccines they had received, and if needed anticipated transplant date and it would give me their individualized accelerated schedule that would be terrifically efficient. [infectious diseases physician] Because most people haven’t seen vaccine-preventable infections like measles or mumps, the diseases aren’t as scary as they should be. Providing a brief blurb about what the disease can look like would make families more inclined to follow through on a vaccine. [transplant nurse coordinator] Having education material about each vaccine would be great—a family could click on it and get a recall of why that vaccine’s important. [hepatologist]
Enhance communication and coordination	Being able to get everyone on the same page to get questions answered would be great. [parent] An app sounds wonderful—if there could be communication between me, my primary care provider, and the transplant team. Everyone could be on the same track. [parent] It’s exhausting trying to get a hold of someone when you have a question, and you can’t go forward until you reach them. The tool would improve communication a hundredfold. [parent] Efficient communication to everybody sounds pretty great. [primary care provider] Families live on their phone, that is the way to communicate with them. [hepatologist]
Centralize vaccine records	I have my child’s vaccines on a card, but if I lose that card or forget to write a new vaccine on the card, I don’t know where that information would be. [parent] When you’re dealing with the stress of a super sick kid you can’t remember every detail like when vaccines were given. It would be great just to open up the app. [parent] A health tool could be a repository for immunizations, particularly for those children from out of state or those children with gaps in their records. [primary care provider] Being able to see in real time the vaccine record would be great. [transplant nurse coordinator] Centralization of records would be great because right now they’re in multiple places. [hepatologist]
Help track when vaccines are due	Reminders would be so big—if all of us transplant families could get reminders, we would be able to get immunizations done on time. [parent] If the app could alert not just me but also the doctor’s office that my child had a shot due that would be really helpful. [parent] A reminder on your phone seems simple buts it’s a huge deal for a transplant patient and their family. [parent] Anything that makes it easier for people to remember when a vaccine is due would help us improve immunization rates. [hepatologist]

### Phase 1: User-Centered App Design, Development, and Alpha Testing

A total of 11 stakeholders attended 2 focus groups. There were 5 parents of transplant recipients (1 parent of a heart transplant recipient, 2 parents of liver transplant recipients, and 2 parents of kidney transplant recipients), 2 transplant nurse coordinators, 2 primary care physicians, and 2 transplant subspecialists. Of the 11 stakeholders, 9 (82%) were female.

Overall, the participants were enthusiastic about the idea of using an app to help facilitate immunization delivery in the pretransplant period. The participants gave specific suggestions on the app features they would find helpful, including the ability to use the app on both their phone and desktop computer and the ability to use finger-touch capability or facial recognition to login.

This app would definitely have made things easier when my child was going through transplant.

parent

I think this app is a great idea for caring for kids with complex medical needs.

primary care physician

When asked their preference on information delivery about the timing and safety of vaccines in the pretransplant period, the participants uniformly liked the idea of informational text rather than an informational video or interactive learning session.

I don’t think I would ever have time to watch a video with my kids running around.

parent

Short text bullets of information make it easy to skim through information while you’re waiting for a visit to start.

parent

I would like information about which specific vaccines my child can and can’t receive before and after transplant.

parent

I like text that I can easily reference when I have questions.

hepatologist

When talking about how to enter the child’s prior immunization records into the app to create a centralized vaccine record, the participants gave valuable input. They all recommended a scroll down feature to select the month or year in which each vaccine was given as opposed to a free text entry stating that “a scroll down feature would minimize entry errors.” A few participants suggested “visual recognition” whereby the app could take a picture of the child’s previous vaccine record and then input that information directly into the app’s record. All participants were concerned about the amount of time it would take to enter a complete vaccine record into the app. However, they stated that they would be willing to make the initial time commitment if thereafter they could always be able to email or print a copy of the vaccine record from the app.

I would definitely be willing to spend 30 minutes or even an hour entering my child’s vaccine records if that meant I could have all the records in one place moving forward.

parent

If I could print out the vaccine record from the app it would be great and worth the initial time needed to enter the vaccines.

parent

When discussing the communication tool or chat feature, the parents and providers were excited about the possibility of being able to facilitate communication between the family, the primary care doctor, and the transplant team. Multiple people mentioned that when the primary care provider and the transplant team use different EMRs, it makes communication very difficult. A few providers expressed concern about having an additional patient communication tool that they would need to check and respond to.

As a parent, it would be huge for the primary care physician and the transplant team to talk directly so I don’t have to be the go-between.

parent

My primary care physician and transplant doctor used two different platforms when my daughter went through transplant, and it was very frustrating. This app would have been ideal.

parent

As a primary care physician, we often feel out of the loop when a child is awaiting transplant; this would have helped to close that loop.

primary care physician

I worry a little about now having to check the EMR communication portal and the app.

hepatologist

### Phase 2: Beta Testing

**Figure 3:**
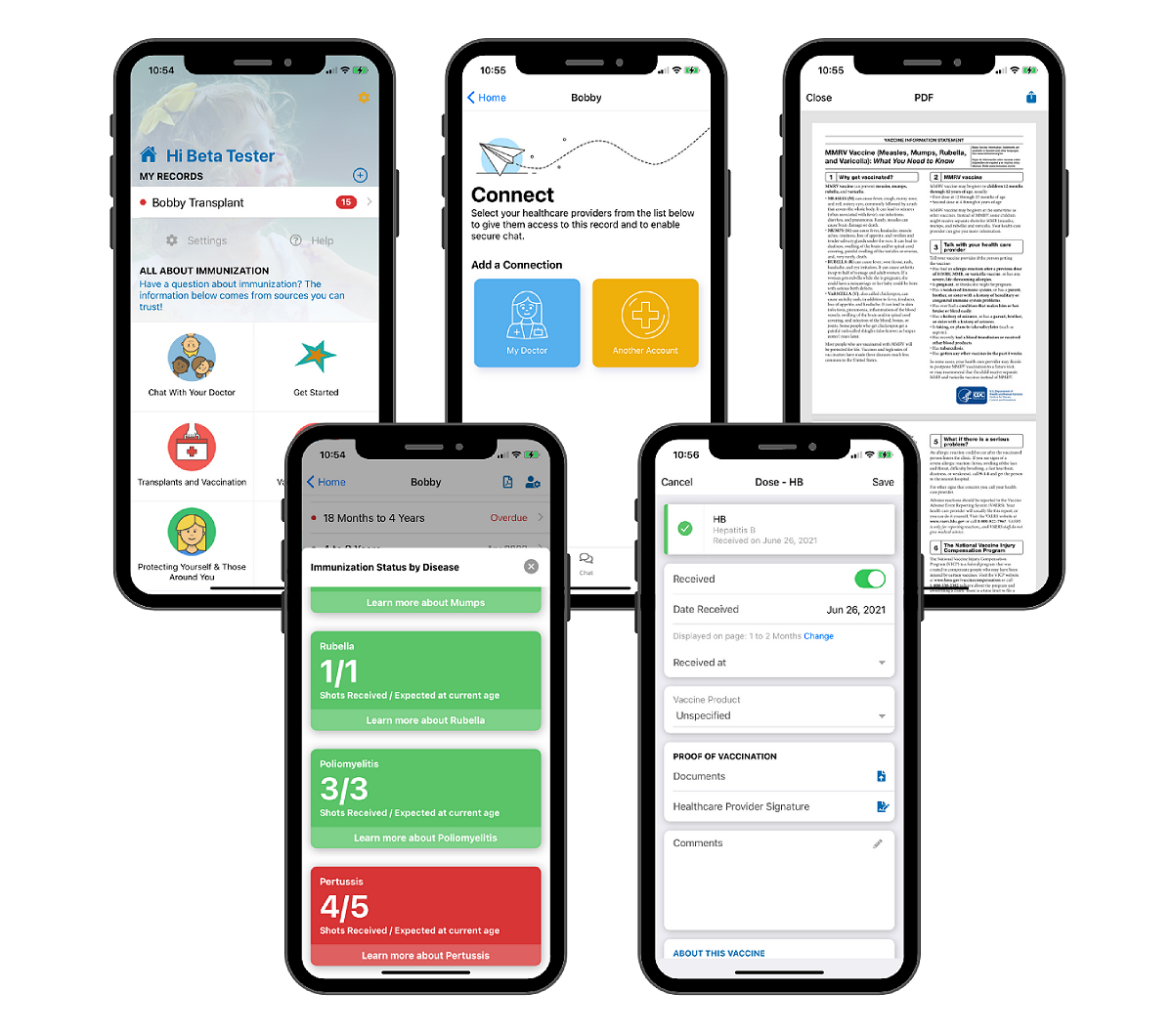
Screenshots from the Immunize PediatricTransplant app.

A total of 12 stakeholders tested the Immunize PediatricTransplant app (10 on an Apple phone and 2 on an Android phone). There were 6 parents of transplant recipients (2 heart transplant recipients, 2 liver transplant recipients, and 2 kidney transplant recipients), 2 transplant nurse coordinators, 2 primary care physicians who had cared for transplant patients, and 2 transplant subspecialists. Of the 12 participants, 10 (83%) were female. User testing identified 6 issues related to usability and functionality. Four usability issues were identified including changing the appearance of the home page to make icons more prominent, changing the specific icon for the chat or communication function, changing the appearance of vaccine information sheets to be more readable, and allowing users to bulk enter vaccines given on the same date or enter multiple dates for the same vaccine. In terms of functionality, the participants suggested 2 features: (1) the inclusion of a PDF tutorial on how to use the app; and (2) the placement of priority stars next to the most administered vaccines in the vaccine entry section in order to help the parents easily identify likely vaccines for each age. Through iterative app changes after every 3-4 user walk-through trials, we modified the app to address all of these issues (Figure 3).

Time stamps from the database identified that, on average, it took 8.1 minutes (SD 1.8 minutes) for the 12 users to enter a 16-vaccine immunization record. The participants entered the vaccine record with 87% (14/16) accuracy (range 69%-100%). All participants were able to connect successfully with the research physician.

Overall, the participants were enthusiastic about the app. All participants stated that the app had the potential to increase knowledge about vaccines and VPIs and improve immunization delivery in the pretransplant period.

Overall, the app is excellent. It’s a very easy to use app…you don’t have to be tech-savvy to use this app.

parent

I think this app would be great not just for transplant patients but all children I care for who require care by multiple subspecialists.

primary care provider

All 12 participants completed the uMARS after they finished usability testing of the app. The average scores for engagement, functionality, aesthetics, and information were 4.2/5, 4.5/5, 4.6/5 and 4.8/5 respectively. Moreover, 100% of the participants reported that they would likely or definitely recommend this app to everyone.

## Discussion

### Principal Findings

In this paper, we described the initial design and user-centered iterative development Immunize PediatricTransplant, a mobile health app designed to increase immunization rates in the pediatric transplant population. Immunize PediatricTransplant addresses and attempts to overcome the barriers to pretransplant immunization by providing educational information about vaccines and VPIs, creating a cloud-based central vaccine record that is easily accessible to families and all members of the health care team regardless of which EMR they use, having a chat feature to enable communication between the family and multiple health care providers, and sending automated reminders (based on the accelerated vaccine schedule used for transplant candidates) to remind families and providers when vaccines are due.

While there are other immunization apps on the market that are able to store a vaccine record and provide general information about vaccines (ie, Docket Immunization Records app [Docket Health Inc], Passport Health, Apple Health app [Apple Inc]), Immunize PediatricTransplant is unique for multiple reasons. First, to our knowledge Immunize PediatricTransplant is the first app that incorporates a communication tool so that multiple providers can communicate directly with the family and other providers about vaccines. Second, Immunize PediatricTransplant is the first app to include an automated vaccine reminder tool that sends out vaccine reminders using a unique vaccine schedule (the accelerated pretransplant schedule). Studies have shown that recall reminders are effective in improving immunization rates regardless of patient age, setting, or vaccination type [32,33]. Additionally, a meta-analysis of 13 randomized controlled studies showed that digital push technologies are more likely to impact vaccine uptake than nondigital interventions [34]. Third, Immunize PediatricTransplant is unique in providing tailored vaccine information for children with a specific health condition (transplant) who require a unique vaccine schedule (the accelerated schedule). The transplant community is a highly motivated and engaged user group that is ideal for pilot testing an mHealth intervention. Fourth, Immunize PediatricTransplant creates a patient-centered cloud-based personal health record across health care networks. Personal health records, which put consumers in control of their health information, are a key feature in health information exchange [10]. If successful in future pragmatic trials, the app could be modified and used not just for transplant candidates and recipients, but also for children with other diseases who have low vaccine rates despite being at high risk for infectious complications (eg, inflammatory bowel disease, rheumatoid arthritis, and lupus) [35-43]. Overall, the participants were extremely positive in their review of the app with 100% of stakeholders recommending the app for transplant families and providers. However, they did express that input of a child’s vaccine record could be improved in future iterations of the app. Currently, the app relies on either the parent or member of the health care team manually entering the name and date of all prior vaccines that the child has received. This leaves potential for error. In this pilot study, 87% of vaccines were entered correctly. Although 7/12 participants (58%) entered the information regarding vaccines with 100% accuracy, there was one participant who had difficulty and only entered 69% of the information correctly. The participants suggested that in the future it would be ideal if one could take a photograph of their child’s prior vaccine record with their phone’s camera, upload the photograph into the Immunize PediatricTransplant app, and then have the app automatically recognize vaccine names and dates. Optimal character recognition with natural language processing has recently been shown to have the potential to accurately identify clinically relevant information contained within the EMR [44-47].

Alternatively, a participant suggested that future iterations of the app could download vaccine information directly from the EMR or state immunization information systems, a feature that has been highlighted as an important future direction in mHealth [10]. However, there is no uniform EMR utilized across health systems in the United States. As of 2017, there were over 600 health information technology developers supplying certified health information technology [48]. In addition, many primary care providers and transplant teams who comanage transplant patients are on different EMRs. Likewise, if a child received vaccines at multiple locations (eg, at the primary care physician’s office, at a community pharmacy, and at their transplant physician’s office), vaccine information may exist in pieces across different EMRs. Unfortunately, state immunization information systems are only accessible by certain in-state providers (not all specialists have access), and transplant patients are often cared for by out-of-state transplant centers. The Immunize PediatricTransplant app allows for all providers, regardless of EMR, to access the complete vaccine record, receive vaccine reminders, and communicate easily with all members of the child’s transplant team.

Certain participants voiced concern that the app could add to the workload of the providers; that it would be another tool that the providers would be responsible for checking and responding to. In future trials of the app among children awaiting liver, heart, and kidney transplant, survey questions will be designed to further understand whether the app helps facilitate communication between various providers and families or adds additional work burden to the providers.

### Strengths

The development of the Immunize PediatricTransplant app was strengthened by the use of various qualitative research techniques (semistructured interviews, focus group discussions, and lab usability think-aloud testing) to ensure that we had a thorough understanding of the pretransplant immunization process including all potential barriers faced by transplant candidates and their providers. A user-centered design at all stages of the development ensured that the app would meet the needs and preferences of all various transplant stakeholders (parents, primary care providers, transplant nurses, and transplant physicians). Finally, the uMARS survey data and data entered into the app’s database complemented the qualitative data to ensure that the app was feasible and acceptable to all transplant stakeholder groups.

### Limitations

Several limitations are potentially present in this pilot study of the Immunize PediatricTransplant app. First, the app was designed and trialed by English speaking transplant stakeholders from 3 large pediatric transplant centers (CHOP, Lurie, and CHCO). As a result, acceptability, feasibility, and usability findings may not be generalizable to all transplant providers and families. Second, participation in the study was voluntary; therefore, it is possible that there was participant bias the whereby providers and parents with enthusiasm for mHealth might have been more likely to participate. These individuals may also have above-average technical skills. Third, some app features (such as the chat or communication feature, vaccine reminder notifications, and the outbreak map) were unable to be fully tested during the beta testing since they require a live app environment. In a future study, we plan to trial this app, including the aforementioned features, in real time among parents and providers with a child currently awaiting heart, liver, and kidney transplant. Finally, the app is dependent on the manual entry of immunizations by the family or provider. If vaccine entry is incomplete or incorrectly entered, then individualized vaccine recommendations may be inaccurate. In a future study trialing the app among families awaiting transplant, we plan to assess the degree of accuracy in parental or provider vaccine entry.

### Conclusions and Future Directions

Despite the high risk for infection posttransplant, the majority of pediatric transplant recipients are underimmunized at the time of transplant. A novel app, Immunize PediatricTransplant, has now been developed, which may overcome the barriers to pretransplant immunization including providing knowledge about vaccines, a communication portal, an easily accessible vaccine record, and an automated vaccine reminder system. This developmental study suggests that the app is functional and acceptable to transplant stakeholders. Future randomized clinical trials among all pediatric solid organ transplant candidates across the United States (agile development phase 3) are needed to trial the app in real time to see if it can improve vaccine rates at the time of transplantation. Additionally, future clinical trials will allow us to evaluate whether the app is useful in educating families about novel vaccines (such as COVID-19) in the pediatric population.

## Abbreviations

CHCO: Children’s Hospital Colorado
CHOP: Children’s Hospital of Philadelphia
EMR: electronic medical record
mHealth: mobile health
uMARS: user version of the Mobile Application Rating Scale
VPIs: vaccine-preventable infections
